# Histopathological features in the clinical specimens with tuberculosis diagnosis by BACTEC MGIT 960 culture

**DOI:** 10.1016/j.jctube.2023.100401

**Published:** 2023-09-29

**Authors:** Nguyen Kim Cuong, Dang Van Thanh, Dinh Van Luong, Nguyen Manh The, Truong Duc Thai, Anh Tran Thi Tuan, Doan Thu Ha, Vu Quoc Dat

**Affiliations:** a Department of Respiratory Tuberculosis, National Lung Hospital, Hanoi, Vietnam; b Department of Tuberculosis and Lung Diseases, Hanoi Medical University, Hanoi, Vietnam; c Department of Infectious Diseases, Hanoi Medical University, Hanoi, Vietnam; d Centre of Lung Transplantation, National Lung Hospital, Hanoi, Vietnam; e Department of Pathology, National Lung Hospital, Hanoi, Vietnam; f Department of Microbiology and National Tuberculosis Reference Laboratory, National Lung Hospital, Hanoi, Vietnam; g Department of Infectious Diseases, Hanoi Medical University, Hanoi, Vietnam; h Hanoi Medical University Hospital, Hanoi Medical University, Hanoi, Vietnam

**Keywords:** Mycobacterium tuberculosis, Histopathology, MGIT culture, Extrapulmonary tuberculosis

## Abstract

Diagnosis of extrapulmonary tuberculosis remains challenging in lower-middle income countries with high burden of tuberculosis (TB). This study aims to describe the histological characteristics in biopsy samples from patients with confirmed TB. This is a retrospective study of clinical biopsy specimens with positive liquid medium culture for *Mycobacterium tuberculosis* and histopathological examination in the National Lung Hospital in Vietnam. Among 1045 biopsy specimens with mycobacteria culture, the overall rate of growth of Mycobacteria tuberculosis in culture was 20.7% (216/1045). The positivity rates of MIGT culture among surgical biopsy specimens were 75% in bone specimen, followed by vertebral specimens (51.3%), and joint specimens (26.4%). For specimens obtained by the fine needle aspiration, the positivity rates of MIGT culture were 26.3% in lymph node and 25.3% in pleural specimen. Among specimens with culture confirmation of TB, the most common histopathoglogical suggestive finding of TB was the presence of epithelioid cell (83.3%), Langhans giant cells (75.9%), and caseous necrosis (75.5%). The high proportion of histological features suggestive of TB among the TB culture confirmed biopsy samples support for further evaluation of histological examination and its combination with other recommended rapid molecular assays in specimens with suspicion of TB.

## 1 Background

Tuberculosis (TB) is the leading cause of death in low- and middle-income countries with an estimated of 1.4 million positive-HIV people deaths and 187,000 deaths among negative-HIV people in 2021[1]. There is a significant fall in the number of people newly TB diagnosed and reported worldwide, from 7.1 million in 2019 to 5.8 million in 2020 – the level last seen in 2012, a significant gap from the approximately 10 million people estimated to developed TB in 2020[1]. Declines in the reported number of TB-infected people in 2020 and 2021 suggest that the number of undiagnosed and untreated people has increased, leading to a growing number of TB deaths and infection transmission [1]. Therefore, the more people develop TB, the more TB-related deaths occur and vice versa. The immediate priority is restoring access to and provision of essential TB services so that levels of TB case detection and treatment can recover to at least 2019 levels, especially in the most badly-affected countries [1].

Vietnam has made a significant achievement in TB control in the past 20 years. The mean delay in TB diagnosis in Vietnam decreased from 13.3 weeks in 1999[2] to 4 weeks in 2007[3]. However, Vietnam still ranked 10th in the top 30 countries suffering the highest burden of TB [1] and the diagnosis remained challenging, especially for extrapulmonary TB. In the second national TB prevalence survey between October 2017 and February 2018, among patients with bacteriologically confirmed TB by an intensified TB pulmonary screening (using TB symptom screening questionnaire, chest X-ray, Ziehl-Neelsen direct light microscopy, Xpert MTB/RIF and BACTEC MGIT 960 liquid culture and Löwenstein-Jensen solid cultures), 32.1% patients were asymptomatic of TB (95 %CI: 26.3–38.6). The laboratory diagnosis of extrapulmonary TB (EPTB) is even more difficult when the patients have no any sputum and mostly depends on rapid molecular test, suggestive histological findings rather than microscopic examination or culture [4]. WHO recommended Xpert MTB/RIF and Xpert Ultra as initial tests in patients with suspicion of extrapulmonary TB for lymph node aspirate, lymph node biopsy, pleural fluid, peritoneal fluid, pericardial fluid, synovial fluid or urine specimens [5]. Samples collected during surgery or biopsy usually undergo histological examination. The understanding of the histological patterns of TB can support clinical judgement when the results of the microbiological tests are undetermined. This study aims to describe the histological pattern suggestiveness of EPTB among TB cultured confirmed clinical samples.

## 2 Methods

This was a retrospective study in clinical biopsy specimens received histopathological examination and culture for mycobacterium species between January 2020 and June 2020 in National Lung Hospital (NLH) in Hanoi, Vietnam. NLH is a tertiary, specialty hospital that dedicated to Tb and lung diseases, under the Ministry of Health and responsible for coordinating and managing TB control activities in Vietnam. The clinical biopsy specimens that were collected by surgical removal or by fine-needle aspiration were sent for to the National Tuberculosis Reference Laboratory that is hosted by NLH for culture using automated Bactec MGIT 960 or 320 system.

We screened all clinical biopsy samples with positive liquid medium culture using automated Bactec MGIT 960 or 320 system and excluded damaged culture samples and cancer-related samples.

A basic demographics (sex and age), site of biopsy, descriptions of histopathological examination and results of culture were extracted from the medical notes. The routine workflow was performed prospectively in a blinded manner in which the culture results were not available to the histopathology personnel. Following the routine procedure at the National Lung Hospital, collected specimen was divided into different parts corresponding to number of testings. One portion was fixed in 10% formalin, embedded in paraffin wax, and subjected to histopathological examination and ZN staining. The samples were examined and evaluated objectively through routine testing HE staining by two histologists. The histopathological patterns considered suggestive of TB required a cyst filled by granulomatous inflammation with central caseous necrosis, Langhans giant cells, epithelioid cells, surrounded by lymphocytes and a peripheral fibrotic edge. The second portion was processed for liquid medium culture. Biopsy samples were inoculated into the BACTEC MGIT 960 and BACTEC 460 at 35 ± 2 °C and examined daily until the end of 42-day incubation. The positive liquid medium culture was tested using BD MGIT TBc Identification Test (TBc ID) test for the identification of *M. tuberculosis*.

Statistical analysis was performed using the IBM SPSS Statistics for Windows, Version 20.0 (Armonk, NY: IBM Corp). The statistical differences between categorical variables were evaluated by the Chi-squared and Fisher's exact tests as appropriated. P value < 0.05 was considered statistically significant.

This study was approved by the Ethical Committee in the National Lung Hospital (No 1056/QD-BVPTU).

## 3 Results

**Fig. 1:**
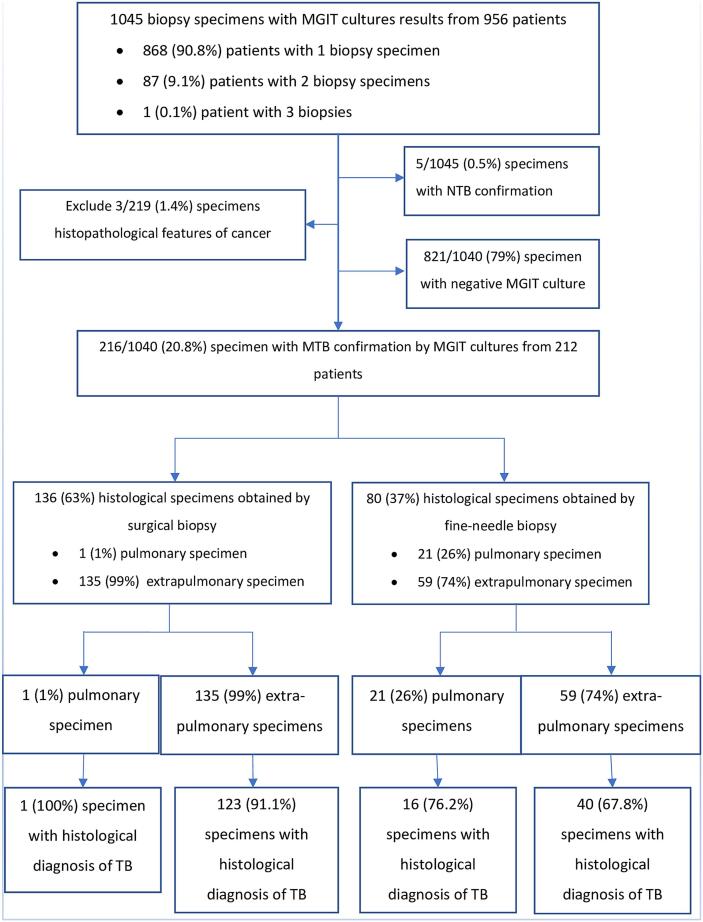
Flowchart of sample collection.

Between January 2020 and June 2020, there were 956 patients with 1045 biopsy specimens requesting MGIT culture. The overall rate of positive culture for *Mycobacterium tuberculosis* was 20.7% (216/1045) and for non-Tuberculous Mycobacteria was 0.5% (5/1045). Among 216 eligible specimens with confirmatory culture for *Mycobacterium tuberculosis*, 136 (63%) specimens were collected surgical biopsy and 80 (37%) specimens were collected by fine-needle biopsy. The detailed number of biopsy specimens was showed in the Fig. 1.

### 3.1 Distribution of MGIT culture results by site of biopsy specimens

**Fig. 2:**
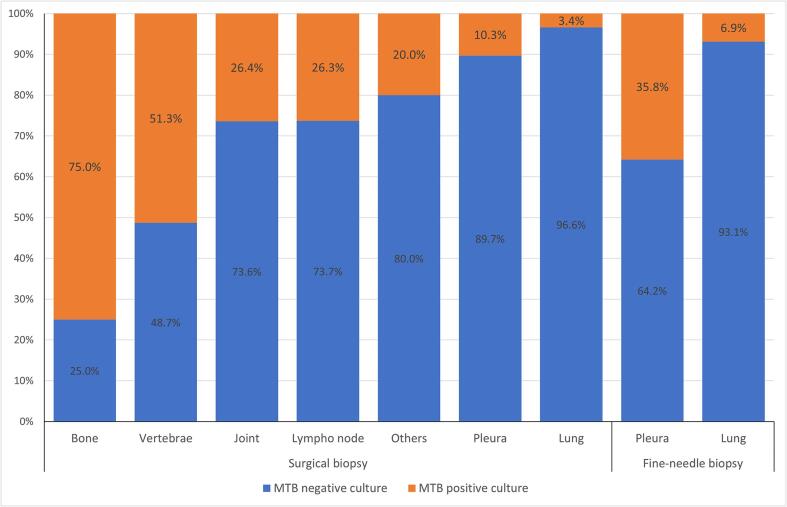
Percentage of MTB positive and negative culture results by sample locations and methods of biopsy.

**Fig. 3:**
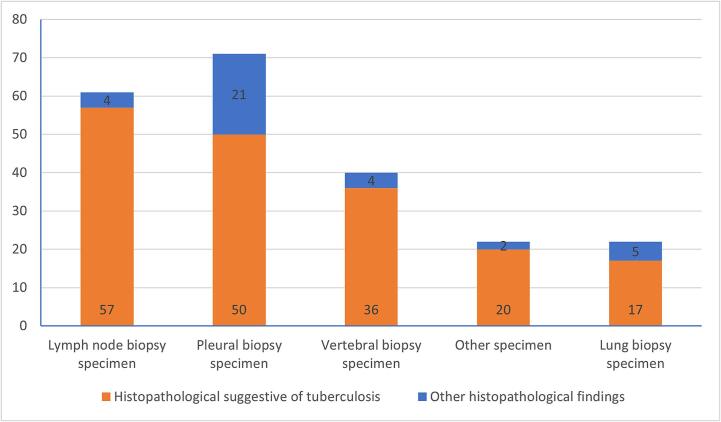
Number of MTB culture confirmed specimens with histological suggestive of tuberculosis.

The most common biopsy specimens were in lung (36.3%), following by pleura (27%) and lymph (22.3%). 526/1040 (50.6%) specimens were collected by surgery and 514 specimens was obtained by fine-needle biopsies. Among specimens obtained by surgery, the positivity rate of MGIT culture was highest in the specimens of bone and spine (6/8 or 75% and 40/78 or 51.3%, respectively) and lowest in the lung specimens. The average rate of positive culture for MTB in surgical specimens was 25.9% (136/526) and it was higher than this rate in fine needle biopsy specimens (15.6%, 80/514) (p < 0.05). The fine-needle pleural biopsies had significantly higher positivity rate of TB culture than surgical biopsies for corresponding specimens (p < 0.05), while those from lung by fine-needle had no higher significance than by surgery (p = 0.71) (Fig. 2).Fig. 3..

### 3.2 Distribution of histopathological TB lesions

Among 216 specimens with MTB confirmation, histopathologically diagnosed TB was made in 83.3% (180/216) and the most common histopathoglogical finding was the presence of epithelioid cell (83.3%), Langhans giant cells (75.9%), and caseous necrosis (75.5%) (Table 1). The proportion of specimens with histopathological findings of TB was highest in lymph node (57/61 or 93.4%), followed by vertebral (36/40 or 90%), lung (17/22 or 77.3%) and pleural specimens (50/71 or 70.4%) (Table 2). The frequency of TB suggestive histological findings among clinical biopsy specimens was showed in table 1 and 2. The examples of histological patterns of TB lesions were presented in Supplementary Figure S1.

**Table 1:** Frequency of TB suggestive histological findings among MTB culture confirmed specimen by mode of biopsy.

Histological pattern	All specimens N = 216	Surgical biopsy n = 136	Fine needle biopsy n = 80	P value
Epithelioid cells	83.3% (180/216)	90.4% (123/136)	71.3% (57/80)	P < 0.001
Langhans giant cells	75.9% (164/216)	85.3% (116/136)	60% (48/80)	P < 0.001
Caseous necrosis	75.5% (163/216)	85.3% (116/136)	58.8% (47/80)	P < 0.001
Other necrosis	64.4% (139/216)	74.3% (101/136)	47.5% (38/80)	P < 0.001
Granulomatous inflammation tissue	47.7% (103/216)	57.4% (78/136)	31.3% (25/80)	P < 0.001
Fibrous tissue	41.7% (90/216)	24.3% (33/136)	71.3% (57/80)	P < 0.00001
Acute inflamed cells	39.8% (86/216)	16.2% (22/136)	80% (64/80)	P < 0.00001
Chronic inflamed cells	1.4% (3/216)	0.7% (1/136)	2.5% (2/80)	P = 0.56

p values were calculated from the comparison between specimens with surgical biopsy and fine needle biopsy.

**Table 2:** Frequency of TB suggestive histological findings among MTB culture confirmed specimen by site of biopsy.

Histological pattern	Pleural biopsy specimen n = 71	Lung biopsy specimen n = 22	Vertebral specimen n = 40	Lymph node specimen n = 61	Other specimens n = 22
Epithelioid cells (n = 180)	74.6% (53/71)	81.8% (18/22)	90% (36/40)	91.8% (56/61)	77.3% (17/22)
Langhans giant cells (n = 164)	64.8% (46/71)	63.6% (14/22)	85% (34/40)	88.5% (54/61)	72.7% (16/22)
Caseous necrosis (n = 163)	56.3% (40/71)	77.3% (17/22)	90% (36/40)	88.5% (54/61)	72.7% (16/22)
Unspecified necrosis (n = 139)	50.7% (36/71)	40.9% (9/22)	62.5% (25/40)	86.9% (53/61)	72.7% (16/22)
Granulomatous inflammation tissue (n = 103)	45.1% (32/71)	22.7% (5/22)	97.5% (39/40)	19.7% (12/61)	68.2% (15/22)
Fibrous tissue (n = 90)	80.3% (57/71)	59.1% (13/22)	37.5% (15/40)	4.9% (3/61)	0.9% (2/22)
Acute inflamed cells (n = 86)	81.7% (58/71)	81.2% (18/22)	10% (4/40)	8.2% (5/61)	4.5% (1/22)
Chronic inflamed cells (n = 3)	0	4.5% (1/22)	0	3.3% (2/61)	0

## 4 Discussion

To our knowledge, this is the large study describing the pattern of histopathological characteristics in diverse biopsy specimens with TB culture confirmation in Vietnam where has a high burden of TB. This study was among few studies focusing on the clinical usefulness of histopathology examination on TB diagnosis. We found that 20.8% of biopsy specimens requesting BACTEC MGIT 960 liquid culture had TB culture confirmation. The highest proportion of positive culture was in surgical biopsy specimen of bone. We described the frequency of histopathological feature characteristic of TB in biopsy specimens. The typical histological findings of tuberculous lesions were observed in 72.7 % TB culture confirmed specimens.

The mycobacterial cultures are time-consuming and usually take 2–6 weeks to yield a result. Because extrapulmonary TB diagnosis remained challenging, the histopathology examination was added to the cultures and molecular testing to improve the diagnosis. In our study, histopathological examination was suggestive of TB in 83.3% of biopsy specimens. This finding was consistent with the previous reports which showed that the TB suggestive histopathology was diagnosed in 89.6% of cases with lymphadenopathy [6] or 72.6% of cases with extrapulmonary TB [7]. However, histological examination was unable differentiate between MTB and NTB histology or other diseases. The sensitivity of histological examination varied by the forms of extrapulmonary TB and the forms of diagnostic techniques [8]. However, in a study of 231 patients with culture confirmed TB in the Republic of Korea, the prevalence of TB-specific findings of caseous necrosis or chronic granulomatous inflammation by histological examination was reported as low as 34.8% for both pulmonary and extrapulmonary TB [9].

The Xpert MTB/RIF demonstrates promising results for the detection of extrapulmonary TB, and its simplicity of use makes it appropriate for use in endemic TB regions [10]. In a study of histological examination in the diagnosis of TB lymphadenitis, histopathology had higher sensitivity (93 vs. 62%) but lower specificity (68 vs. 83%) versus Xpert MTB/RIF [11]. The sensitivity of histopathological diagnosis for TB can increase by combining with Xpert MTB/RIF (up to 95%)[12].

Major limitation of our study was a single-center study in a specialized tertiary hospital, the lack of representativeness and generalization. Because histopathology diagnosis is based on the subjective (but trained) opinion of pathologists, hence impairing the study results’ ability to generalize to other settings without relevant expertise and capacity.

In conclusion, we found a high proportion of histological features suggestive of TB among the TB culture confirmed biopsy samples. Further studies need to evaluate the performance of histological examination in specimens with suspicion of TB in the setting with higher burden of TB.

## Declaration of Competing Interest

The authors declare that they have no known competing financial interests or personal relationships that could have appeared to influence the work reported in this paper.
