# Supplementary material for: Histopathological features in the clinical specimens with tuberculosis diagnosis by BACTEC MGIT 960 culture
Source: J Clin Tuberc Other Mycobact Dis. 2023 Sep 29;33:100401. doi: 10.1016/j.jctube.2023.100401 (PMC10622830; doi:10.1016/j.jctube.2023.100401)

Supplementary figure 1. Histopathological features in different biopsy specimens using standard haematoxylin-eosin (H&E) stain (original magnification ×400).

A. Lung specimen: Langhans giant cell (green arrow), epithelioid cell (red arrow) and caseous necrosis (yellow arrow). B Spine specimen: degenerative changes of red blood cells (green arrow), necrosis (red arrow) and osteonecrosis (yellow arrow). C Pleural specimen: Vasculogenesis (green arrow), Epithelioid cell (red arrow) and lymphoid cells (yellow arrow). D Lymph node specimen: Langhans giant cell (green arrow), epithelioid cell (red arrow) and caseous necrosis (yellow arrow).


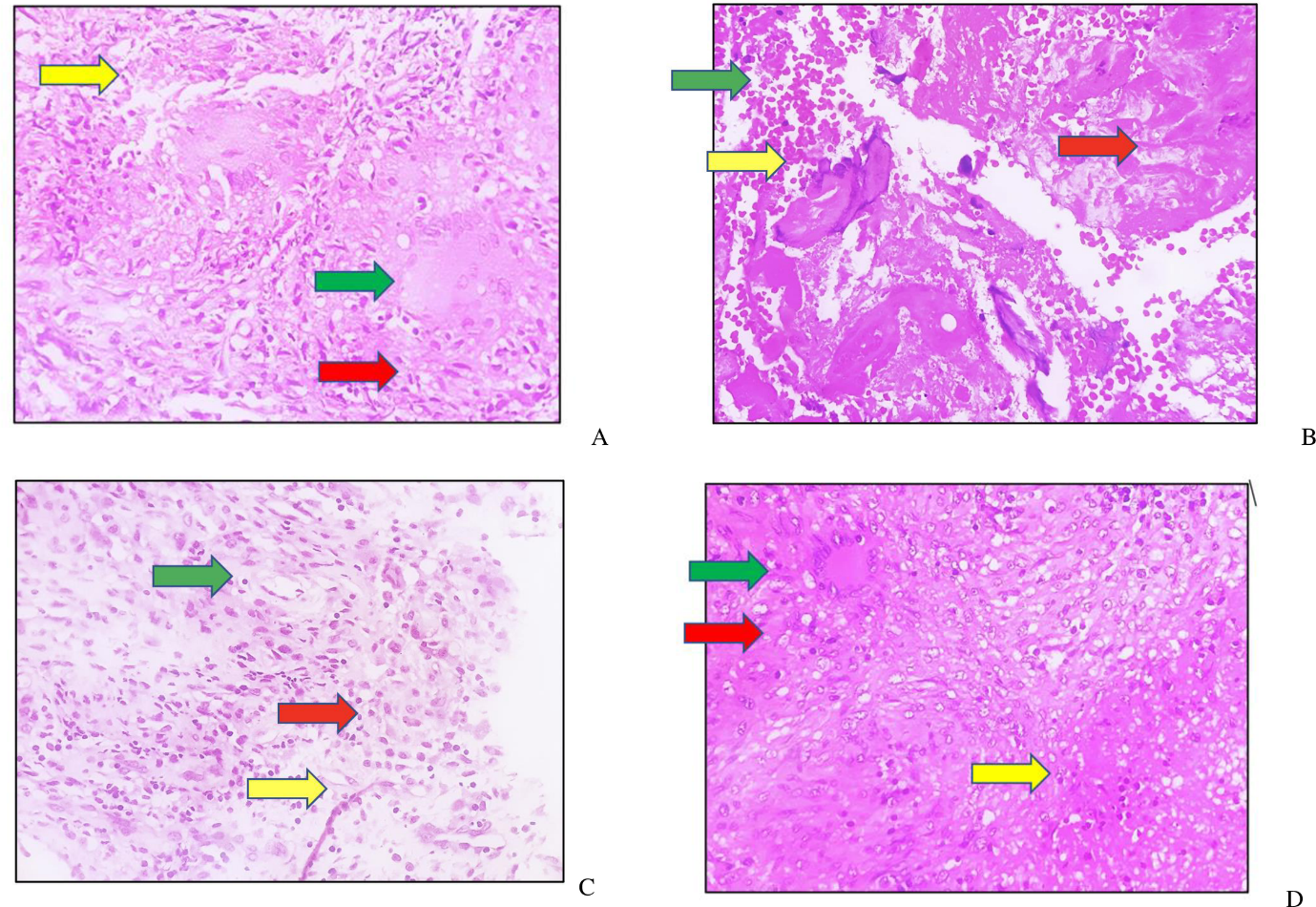

Supplement: Supplementary data 1 [file mmc1.docx]
